# Anatomical Variation of Abdominal Wall Musculature: An Objective Assessment Using Cross-Sectional Imaging

**DOI:** 10.3389/jaws.2024.13114

**Published:** 2024-06-21

**Authors:** Samuel George Parker, Helena Blake, Steve Halligan, Athanasius Ishak, Hossam Mahrous, Mohammed Abdelgelil, Alastair Colin James Windsor, Arun Shanmuganandan, Ravishankar Jakkalasaibaba, Rhys Thomas

**Affiliations:** ^1^ The Abdominal Wall Unit, Croydon University Hospital, London, United Kingdom; ^2^ University College London Centre of Medical Imaging, London, United Kingdom; ^3^ The Abdominal Wall Unit, The Princess Grace Hospital, London, United Kingdom

**Keywords:** hernia, abdominal wall anatomy, computed tomography, reconstruction surgery, variability

## Abstract

**Purpose:** To determine normal anatomical variation of abdominal wall musculature.

**Methods:** A retrospective analysis of CT scans was performed on adults (>18 years) with normal abdominal wall muscles. Two radiologists analysed the images independently. Distances from three fixed points in the midline were measured. The fixed points were; P1, mid-way between xiphoid and umbilicus, P2, at the umbilicus, and P3, mid-way between umbilicus and pubic symphysis. From these three fixed points the following measurements were recorded; midline to lateral innermost border of the abdominal wall musculature, midline to lateral edge of rectus abdominis muscle, and midline to medial edges of all three lateral abdominal wall muscles. To obtain aponeurotic width, rectus abdominis width was subtracted from the distance to medial edge of lateral abdominal wall muscle.

**Results:** Fifty normal CT scan were evaluated from between March 2023 to August 2023. Mean width of external oblique aponeurosis at P1 was 16.2 mm (IQR 9.2 mm to 20.7 mm), at P2 was 23.5 mm (IQR 14 mm to 33 mm), and at P3 no external oblique muscle was visible. Mean width of the internal oblique aponeurosis at P1 was 32.1 mm (IQR 17.5 mm to 45 mm), at P2 was 10.13 (IQR 1 mm to 17.5 mm), and at P3 was 9.2 mm (IQR 3.0 mm to 13.7 mm). Mean width of the transversus abdominis aponeurosis at P1 was −25.1 mm (IQR 37.8 mm to −15.0 mm), at P2 was 29.4 mm (IQR 20 mm to 39.8 mm), and at P3 was 20.3 mm (IQR 12 mm to 29 mm).

**Conclusion:** In this study we describe normal anatomical variation of the abdominal wall muscles. Assessing this variability on the pre-operative CT scans of ventral hernia patients allows for detailed operative planning and decision making.

## Introduction

Abdominal Wall Reconstruction (AWR) is a growing and developing subspecialty within General Surgery [1]. Over the last 10 years new techniques have evolved to tackle abdominal wall reconstruction, for example, transversus abdominis release [2], peritoneal flap hernioplasty [3], endoscopic anterior component separation [4], and robotic retrorectus repair [5]. Ventral hernia patients are often co-morbid; many are obese, diabetic, and have coronary artery disease. The decision whether or not to operate frequently necessitates discussion within an AWR multidisciplinary team (MDT) meeting with consultant surgeons (plastics and general), radiologists, and anaesthetists [6]. These meetings often focus on patients’ pre-operative CT scans, where hernia morphology is assessed, including the length, thickness, and insertion site of the lateral abdominal wall musculature.

To date there is little guidance regarding which reconstructive technique should be used for any particular hernia, with the technique adopted usually based on surgeon preference, which is influenced heavily by familiarity. During MDT discussions we have noticed that the lateral abdominal wall muscles exhibit varying anatomy, something also observed during surgical procedures. While there are many CT studies analysing ventral hernia morphology [7–9], there are few studies that assess the normal anatomical variation of the abdominal wall musculature. We hypothesize that by studying CT scans of patients with apparently normal abdominal wall anatomy, our understanding of variations of abdominal wall muscles will improve, which will aid pre-operative surgical planning and enhance selection of the most effective reconstructive technique for ventral hernia repair.

## Methods

### Study Design

A single site retrospective observational study was performed to assess anatomical variation of abdominal wall muscles. CT scans with normal abdominal wall anatomy were analysed. Patients were selected using an online random dates generator that produced dates from January 2019 onwards. The first CT abdomen and pelvis with intravenous contrast performed on the date generated was identified and then scrutinised for the inclusion/exclusion criteria as follows: Inclusion criteria: Adult patients (defined as >18 years), without previous abdominal surgery (after review of each participants electronic clinical record) and with a linea alba width of less than 2 cm wide (to exclude divarication) [10]. Exclusion criteria included any abdominal wall hernia visible on CT (except inguinal hernias) and radiological signs of bowel obstruction. All patients were asked, by telephone, whether their CT scan could be used for research purposes and for assessment of their abdominal wall muscles; six patients meeting the inclusion criteria did not want their scan to be analysed and were excluded from the study, all included patients agreed to take part. Ethical approval for this study was obtained from (BLINDED FOR REVIEW) Hospital’s research and development department and the final manuscript was approved for submission and publication. A protocol was written prior to data collection.

### Baseline Characteristics

Baseline demographics and basic medical characteristics were obtained. Each patient’s electronic clinical record was assessed; age, sex, and BMI at the time of CT scanning were extracted. In addition, smoking status as well as a medical history of diabetes, cardiac disease, and COPD were also recorded.

### CT Protocol

All CT scans were performed with the patient supine in suspended respiration, using either one of our two outpatient scanners; either our GE Discovery CT750 HD (General Electric, Buckinghamshire, United Kingdom) or our Canon Aquilion ONE GENESIS Edition (Canon Medical Systems Ltd., West Sussex, United Kingdom). Initially a 20G cannula was inserted and a preliminary scanogram was performed to ensure the scan extends from above the diaphragm to below the lesser trochanter. Thereafter 80mLs of non-ionic iodine-based contrast medium, Omnipaque 300 (General Electric, Buckinghamshire, United Kingdom), was injected at a rate of 3–4 mL/s.

### Data Collection

**FIGURE 1:**
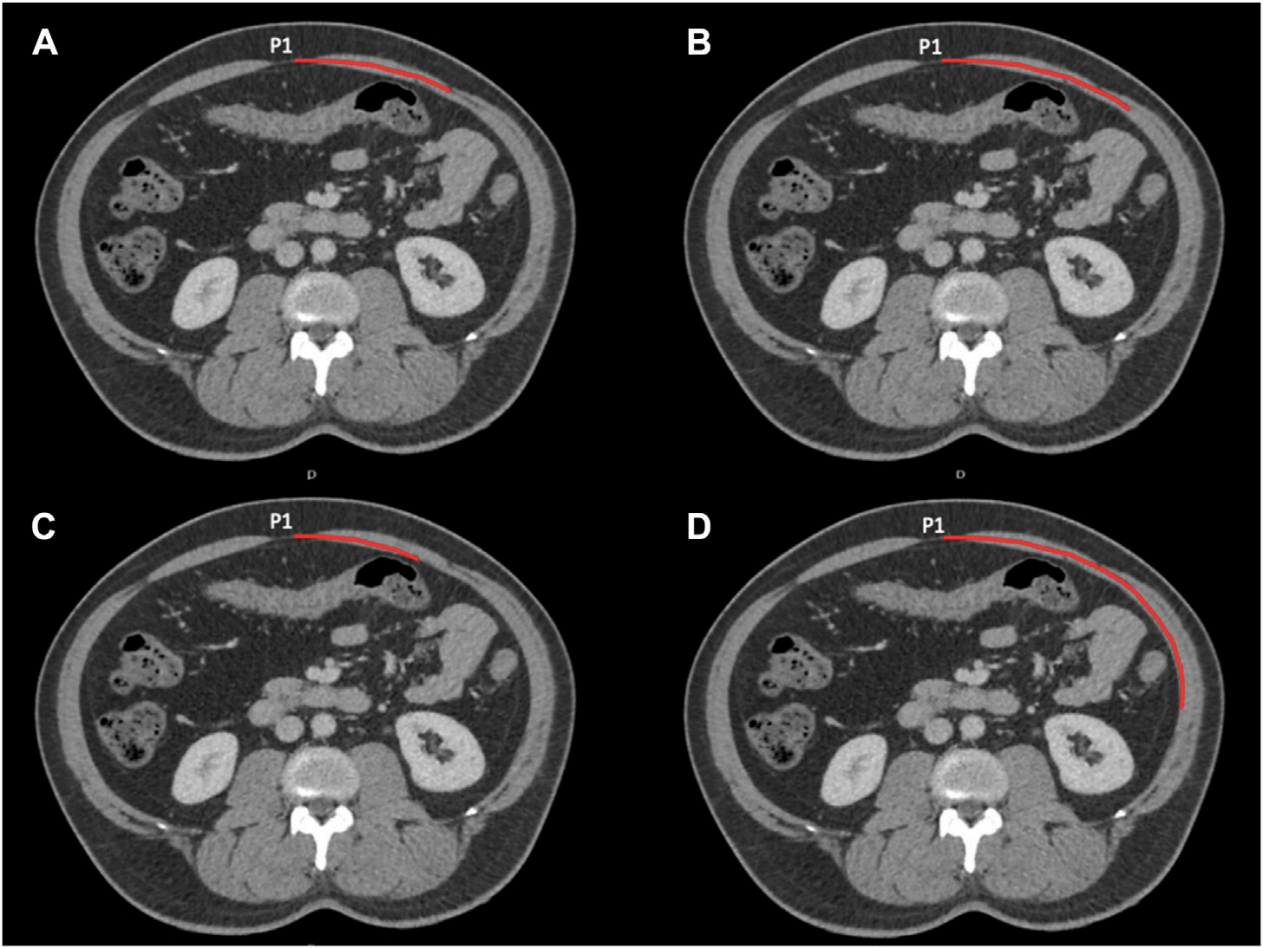
Four axial images at P1 showing the lengths measured. Measurements start in the midline. **(A)** Shows the width of the rectus abdominis and the distance the medial edge of the external oblique (these lengths are the same in this instance). **(B)** The distance to the medial edge of the internal oblique. **(C)** The distance to the medial edge of the transversus abdominis. **(D)** The distance to the lateral most inner point of the lateral abdominal wall musculature.

Two staff grade radiologists each with over 10 years of experience analysed the CT scans (BLINDED FOR REVIEW). Five patients’ scans were evaluated by both radiologists to assess inter-reader agreement; thereafter, scans were assessed independently. Distances were measured using a medical image workstation, Sectra Workstation IDS7, Version 21.2.16.6372 (Sectra AB, Teknikringen 20, SE-583 30 Linkoping, Sweden). All distances/measurements used a curved line parallel to the curvature of the abdominal wall and through the centre of the rectus abdominis muscle, as shown in Figure 1. They were not simply straight-line estimates between two points. The following variables were measured for each participant and recorded in a spreadsheet (Microsoft Excel for Mac version 16.48, Microsoft Corporation).

#### Abdominal Cavity

To evaluate overall abdominal wall dimension, midline abdominal wall length was measured from the diaphragm (at the right cardiophrenic angle) to the superior margin of the symphysis pubis. The maximal width of the abdominal cavity between the two most lateral, inner borders of abdominal wall musculature was also measured. The length of the linea alba was measured from the tip of the xiphoid to the superior edge of the pubis.

To describe anatomical variability, we used three reference points in the midline:

P1—midway point between the xiphoid and umbilicus.

P2—at the umbilicus.

P3—midway point between the umbilicus and pubic symphysis.

#### Rectus Abdominis

Using axial images, on the left we measured half the width of the anterior abdominal wall from the midline point P1 to the most lateral inner border of the lateral abdominal wall musculature (as in Figure 1; measurement D), from P2 to the most lateral inner border of the lateral abdominal wall musculature, and from P3 to the most lateral inner border of the lateral abdominal wall musculature. The length from the midline to the lateral edge of the left rectus abdominis muscle was also measured at levels P1, P2, and P3.

#### External Oblique

Points P1, P2, and P3, were used to measure the distance from the midline to the medial edge of the external oblique muscle. External oblique aponeurosis width was later calculated by subtracting rectus muscle width at each respective point.

#### Internal Oblique

Points P1, P2, and P3, were then used to measure the distance from the midline to the medial edge of the internal oblique muscle. Internal oblique aponeurosis width was later calculated by subtracting rectus muscle width at each respective point.

#### Transversus Abdominis

**FIGURE 2:**
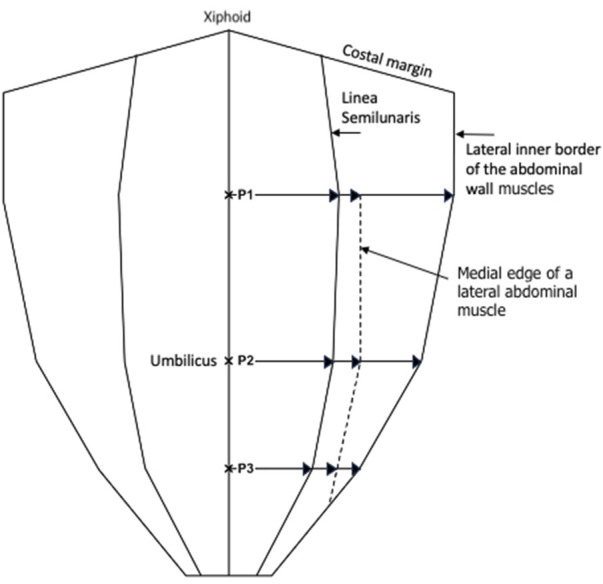
Schematic diagram showing the measurements made at P1, P2, and P3, with P1 being halfway between the xiphoid and umbilicus, P2 being at the umbilicus, and P3 being halfway between the umbilicus and the pubic symphysis.

Transversus abdominis inserts into the posterior rectus sheath medial to the semilunar line^1^ above the arcuate line. Below the arcuate line it inserts into the combined tendon that travels anterior to the rectus abdominus (anterior rectus sheath). Points P1, P2, and P3, were used to measure the distance from the midline to the medial edge of the transversus abdominis muscle. Transversus abdominis aponeurosis width was later calculated by subtracting rectus muscle width at each respective point. Figure 2 is a schematic diagram that describes how these dimensions were calculated.

Axial measurements from P1, P2, and P3 were also expressed as proportions or fractions with each measurement being divided by the corresponding width of the anterior abdominal wall from the midline to the lateral inner border of abdominal wall musculature. This meant that our measurements can be used to describe anatomical variability of both the rectus abdominis muscle and the lateral abdominal wall muscles in all patients.

### Statistics

Baseline characteristics are reported as narrative and simple descriptive statistics. Bland-Altman analysis with 95% limits of agreement were used to assess inter-reader agreement. Anatomical dimensions were reported as means, inter-quartile ranges, and a range from the 10th to 90th centile. Anatomical diagrams were created to display the anatomical variability of the rectus abdominis and all three lateral abdominal wall muscles.

## Results

**FIGURE 3:**
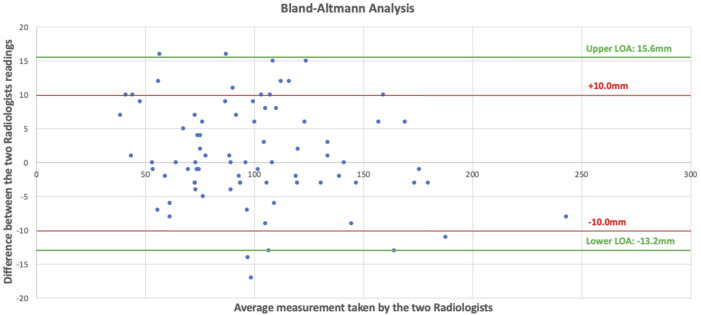
Scattered plot showing our Bland-Altman analysis with differences of the measurements on the y axis and mean of the two radiologists’ measurements on the x axis.

Fifty patient CT scans were evaluated from between March 2023 to August 2023. Radiologists (BLINDED FOR REVIEW) both extracted data from 5 CT scans, allowing for a direct comparison of 80 measurements. Bland-Altman analysis gave upper and lower limits of agreement of 15.6 mm and −13.2 mm respectively. As 80% of the measurements lay within the limits of ±10 mm the readings were deemed as acceptable for this investigative study (Figure 3).

The CT scans analysed were performed for a range of indications. Thirteen were colorectal cancer screening scans, nine were outpatient requests for chronic abdominal pain, eight were performed for unintentional weight loss, eight were emergency scans, four were staging CT scans, two for trauma, for four there was no indication, and for two the indications were miscellaneous. All CT scans had an intact anterior abdominal wall. Thirty-one scans were performed on males. The mean age of scanned participants was 60, with a mean body mass index of 27.04. Nineteen patients were smokers, 20 patients had diabetes, 11 had cardiac disease, and three had COPD. The mean abdominal cavity length was 342.3 mm, with a mean width of 271.9 mm. Average linea alba length was measured as 366.5 mm, with average widths of half of the curvature of the anterior abdominal wall (from the midline to the lateral-most inner border of the abdominal wall musculature) at P1, P2, and P3, being measured as 193.7, 163.9, and 110.6 mm, respectively.

For the rectus abdominis; mean proportional width of the muscle relative to the width of the anterior abdominal wall at P1 was 0.47, (IQR 0.44 to 0.51, 10th centile 0.39, 90th centile 0.54), at P2 was 0.53 (IQR 0.48 to 0.58, 10th centile 0.42, 90th centile 0.68), and at P3 was 0.632 (IQR 0.52 to 0.71, 10th centile 0.48, 90th centile 0.80).

**FIGURE 4:**
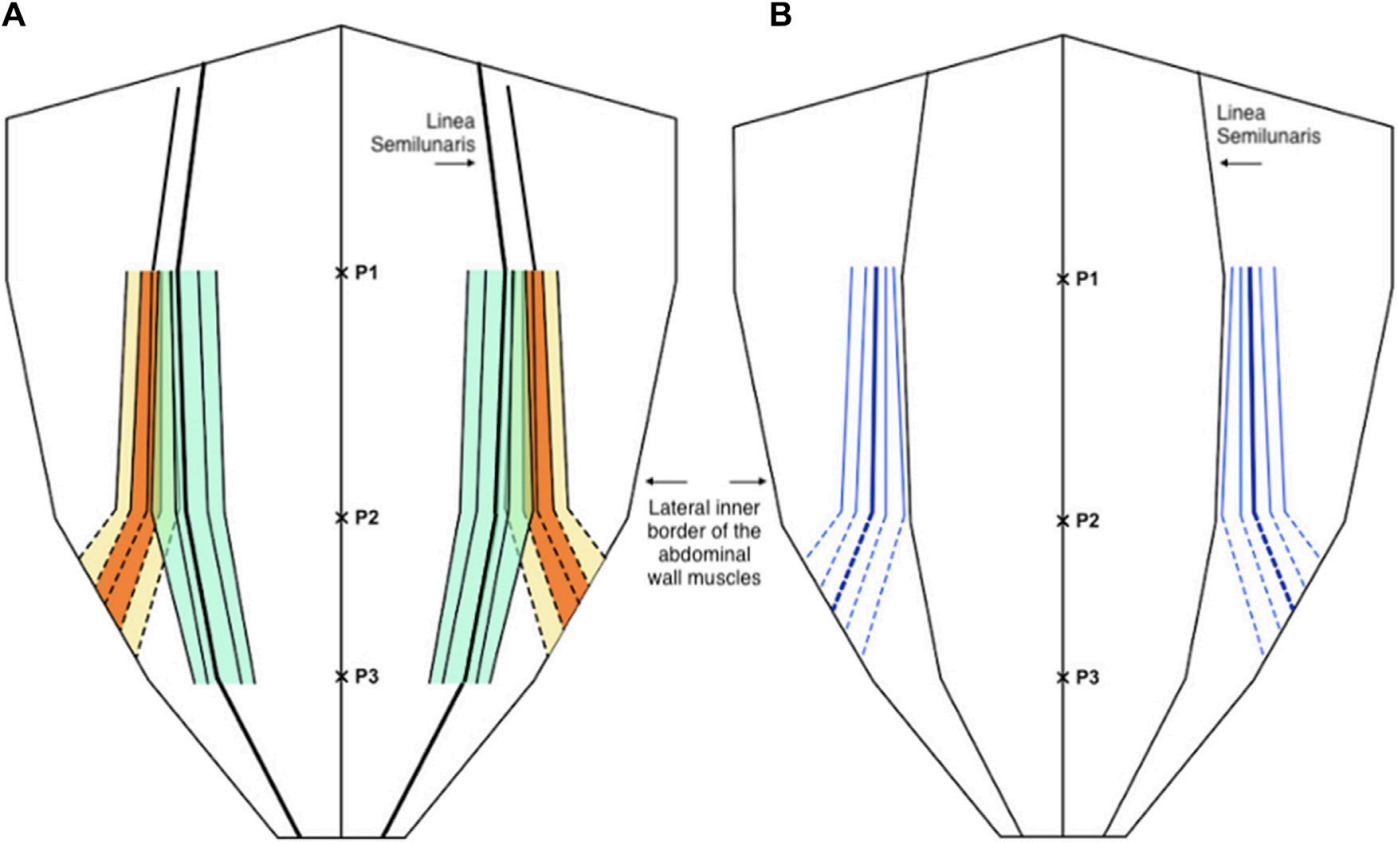
**(A)** Distribution of the medial edge of the external oblique muscle (orange and yellow) and the lateral edge of the rectus abdominis muscle (green) as a proportion (or fraction) of the anterior abdominal wall, below P2 the dotted lines are estimates of the anatomical variability as the external oblique muscle is not present at or below the level of P3. **(B)** The width of the external oblique aponeurosis as a distance from the linea semilunaris (blue), below P2 the dotted lines are estimates of the anatomical variability as the external oblique muscle is not present at or below the level of P3. In both diagrams the central line represents the mean and the parallel lines represent the inter-quartile range (IQR), 25th and 75th centiles, and 10th and 90th centiles.

For the medial edge of external oblique; mean proportional distance from the midline (relative to the width of the anterior abdominal wall) at P1 was 0.56 (IQR 0.51 to 0.59, 10th centile 0.48, 90th centile 0.63), at P2 was 0.67 (IQR 0.6 to 0.74, 10th centile 0.54, 90th centile 0.80), and at P3 it was not possible to take measurements as the muscle had become aponeurosis in 49 out of 50 participants. Mean width of external oblique aponeurosis from the lateral edge of rectus at P1 was 16.2 mm (IQR 9.2 mm to 20.7 mm, 10th centile 5.9, 90th centile 30.1 mm), at P2 was 23.5 mm (IQR 14 mm to 33 mm, 10th centile 5.8 mm, 90th centile 40.4 mm), and at P3 again it was not possible to measure aponeurotic width as the muscle had become aponeurosis at this level (Figure 4).

**FIGURE 5:**
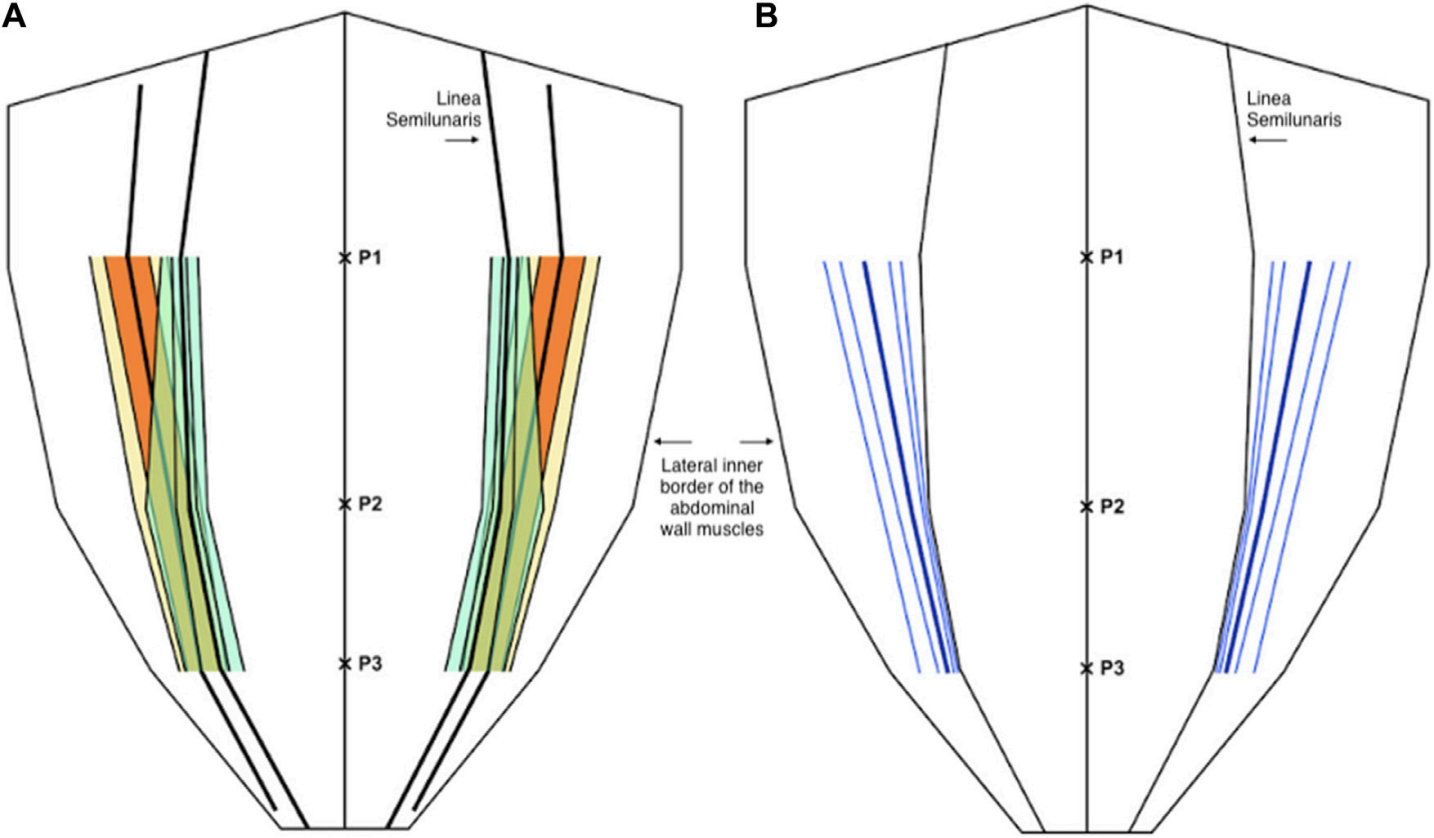
**(A)** Distribution of the medial edge of the internal oblique muscle (orange and yellow) and the lateral edge of the rectus abdominis muscle (green) as a proportion (or fraction) of the anterior abdominal wall. **(B)** The width of the internal oblique aponeurosis as a distance from the linea semilunaris (blue). In both diagrams the central line represents the mean and the parallel lines represent the inter-quartile range (IQR), 25th and 75th centiles, and 10th and 90th centiles.

For the medial edge of the internal oblique; mean proportional distance from the midline (relative to the width of the anterior abdominal wall) at P1 was 0.64 (IQR 0.57 to 0.71, 10th centile 0.52, 90th centile 0.75), at P2 was 0.59 (IQR 0.52 to 0.66, 10th centile 0.49, 90th centile 0.71), and at P3 was 0.71 (IQR 0.65 to 0.77, 10th centile 0.60, 90th centile 0.83). Mean width of the internal oblique aponeurosis from the lateral edge of rectus at P1 was 32.1 mm (IQR 17.5 mm to 45 mm, 10th centile 10.7 mm, 90th centile 54.6 mm), at P2 was 10.13 (IQR 1 mm to 17.5 mm, 10th centile 0.5 mm, 90th centile 27 mm), and at P3 was 9.2 mm (IQR 3.0 mm to 13.7 mm, 10th centile 0.5 mm, 90th centile 25 mm) (Figure 5).

**FIGURE 6:**
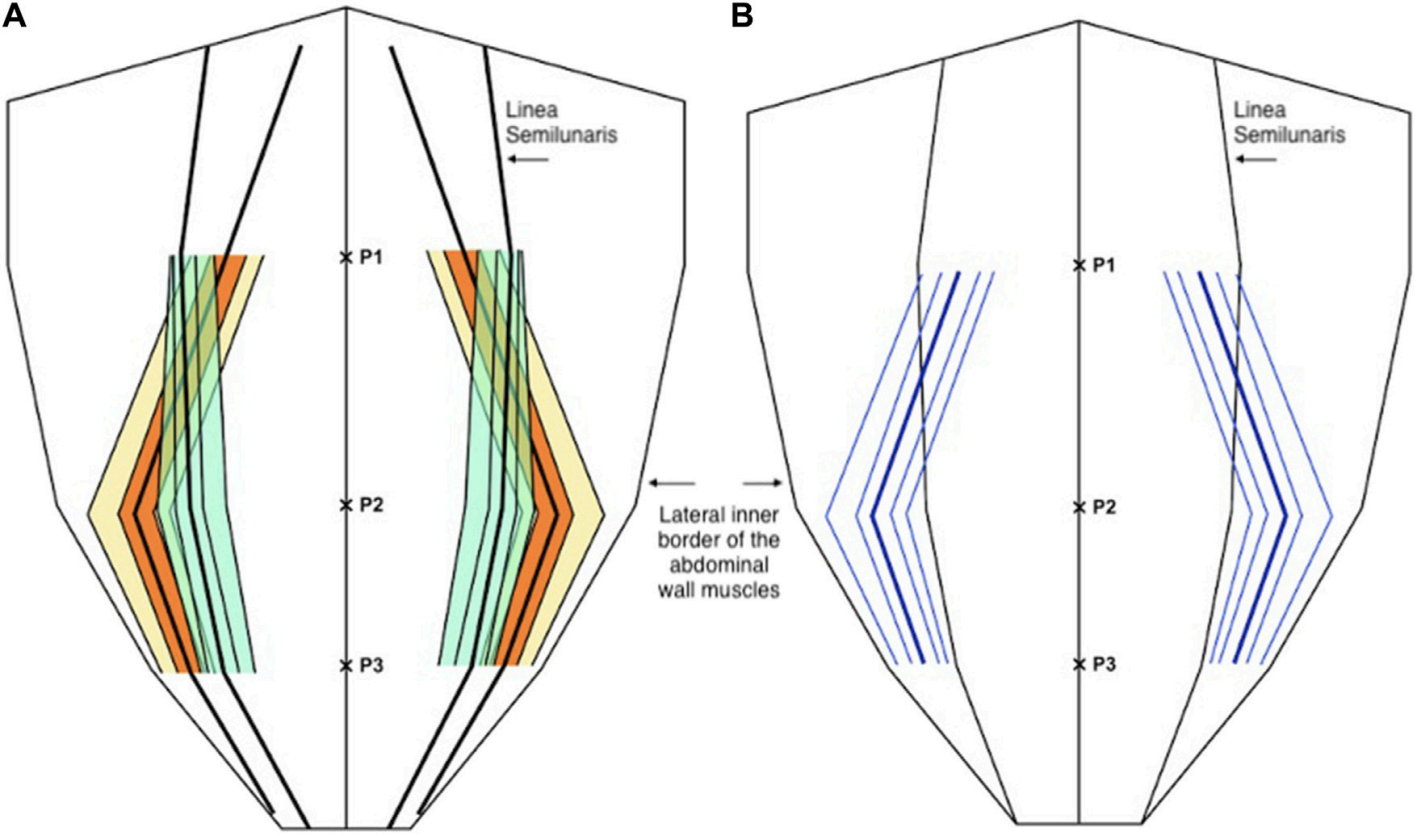
**(A)** Distribution of the medial edge of the transversus abdominis muscle (orange and yellow) and the lateral edge of the rectus abdominis muscle (green) as a proportion (or fraction) of the anterior abdominal wall. **(B)** The distance of the medial edge of the transversus abdominis inserting into the posterior sheath (medial to the linea semilunaris) and the width of the transversus abdominis aponeurosis (lateral to the linea semilunaris) (blue). In both diagrams the central line represents the mean and the parallel lines represent the inter-quartile range (IQR), 25th and 75th centiles, and 10th and 90th centiles.

For the medial edge of the transversus abdominis, mean proportional distance from the midline (relative to the width of the anterior abdominal wall) at P1 was 0.34 (IQR 0.28 to 0.41, 10th centile 0.20, 90th centile 0.47), at P2 was 0.71 (IQR 0.63 to 0.79, 10th centile 0.51, 90th centile 0.87), and at P3 was 0.82 (IQR 0.77 to 0.88, 10th centile 0.67, 90th centile 0.93). Mean width of the transversus abdominis aponeurosis from the lateral edge of rectus at P1 was −25.1 mm (IQR -37.8 mm to −15.0 mm, 10th centile −45.1 mm, 90th centile −2.9 mm) (negative results imply positive overlap of the transversus abdominis with the posterior sheath), at P2 was 29.4 mm (IQR 20 mm–39.8 mm, 10th centile 8.6 mm, 90th centile 57.3 mm), and at P3 was 20.3 mm (IQR 12 mm–29 mm, 10th centile 7.2 mm, 90th centile 35 mm) (Figure 6).

## Discussion

**FIGURE 7:**
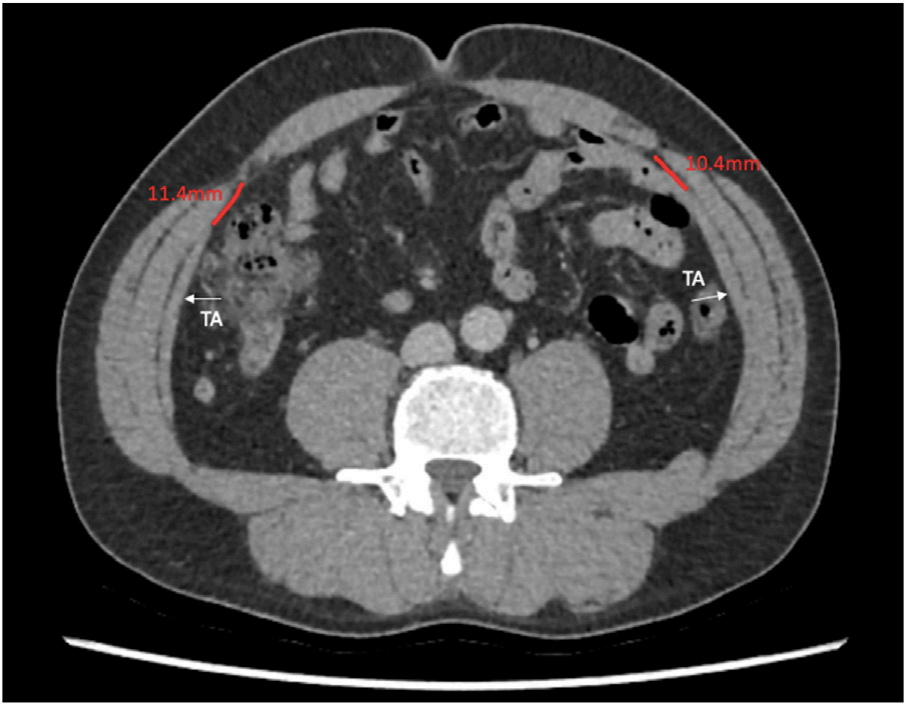
Axial image at the umbilicus showing the transversus abdominis 10.4 and 11.4 mm away from the linea semilunaris.

**FIGURE 8:**
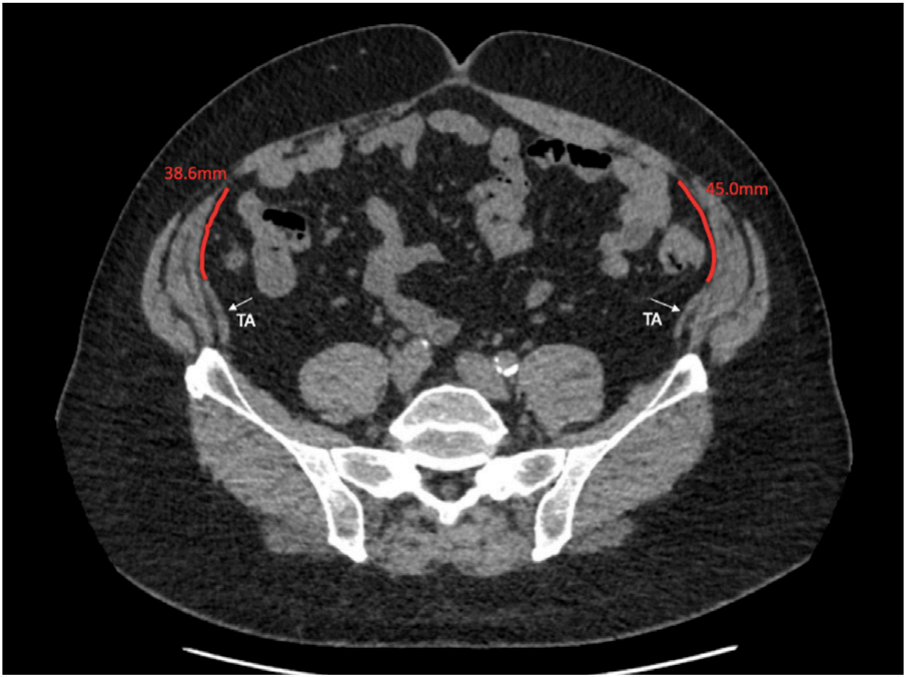
Axial image at the umbilicus showing the transversus abdominus 38.6 and 45.0 mm away from the linea semilunaris.

This study describes the anatomical variation of normal abdominal wall musculature. We believe that anatomical variation is a key surgical concept that abdominal wall surgeons must consider increasingly as the subspecialty evolves. Previous studies of abdominal wall anatomy have demonstrated variation in the level of the arcuate line [11], in anterior abdominal wall cutaneous innervation [12], in the distribution of preperitoneal fat [13], and in the vascular supply to the anterior abdominal wall [14]. We believe that this study is the first to use cross sectional imaging to interrogate the distribution of the abdominal wall muscles and to report their anatomical variation in detail. We found that midway between the xiphoid and the umbilicus, the full range of overlap of the transversus abdominis with the posterior sheath varied from −6 mm to 49 mm, and this variation, along with transversus abdominis length, is highly likely to have an effect on the amount of release gained from posterior component separation. At Croydon, our intra-operative experience is that after preforming a posterior component separation on a medial transversus abdominis muscle and after developing the pre-transversalis or pre-peritoneal plane you get much more advancement of the posterior sheath. We suspect that this is due to a greater length of muscle being released that was previously retracting the sheath away from the midline. We are currently working on further work that investigates this in greater depth. Our theory is similar to colorectal surgery where the length of sigmoid colon affects a colorectal surgeon’s ability to achieve a tension free colorectal anastomosis, we hypothesise that the amount of transversus abdominis overlap with the posterior sheath affects an abdominal wall surgeon’s ability to achieve tension free midline closure. We also found considerable variation in the length of the transversus abdominis aponeurosis at the level of the umbilicus. Since many incisional hernias originate from at or around the umbilicus, anatomical variability here will facilitate prediction of the amount of release that can be gained from a posterior component separation (Figures 7, 8).

Our findings are not solely limited to the distribution of transversus abdominis. The distribution of the external oblique is also described, with nearly all patients demonstrating no muscle remaining below the level of P3, the midway point between the umbilicus and the pubic symphysis. This large aponeurotic sheet spans the lower quarter of the anterior abdominal wall and could be used in future reconstructive surgery. Furthermore, in our unit many flank incisional hernias seem to result from defects in the transversus abdominis and internal oblique, with the more superficial external oblique aponeurosis remaining intact. These “inter-oblique hernias” [15] may result from internal oblique and transversus abdominis muscle ischaemia at the time of closing oblique abdominal wall incisions, with the relatively avascular external oblique aponeurosis being less vulnerable to ischaemia, less vulnerable to impaired wound healing, and consequently less likely to generate a defect. The distribution of the internal oblique is also described, with its aponeurosis remaining thin throughout the length of the semilunar line. We also observed that the internal oblique was, in most cases, the thickest muscle of the lateral abdominal wall, although we did not measure this. Being the longest and thickest muscle, we conclude that it is could be the “workhorse” of the abdominal wall and crucial in maintaining strength and integrity. Further work is required to analyse each abdominal wall muscles effect on truncal stability and biomechanical strength.

**FIGURE 9:**
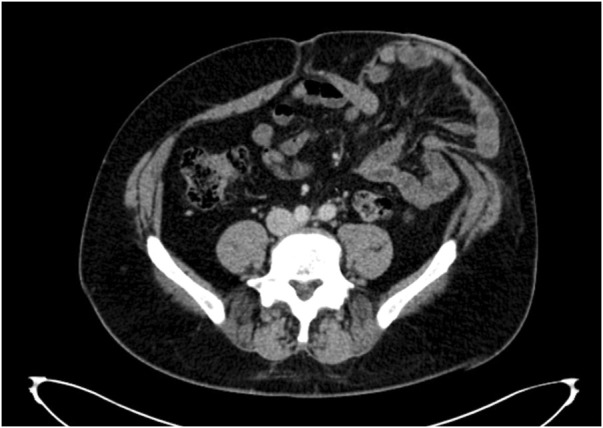
Axial image through the centre of a parastomal hernia defect.

**FIGURE 10:**
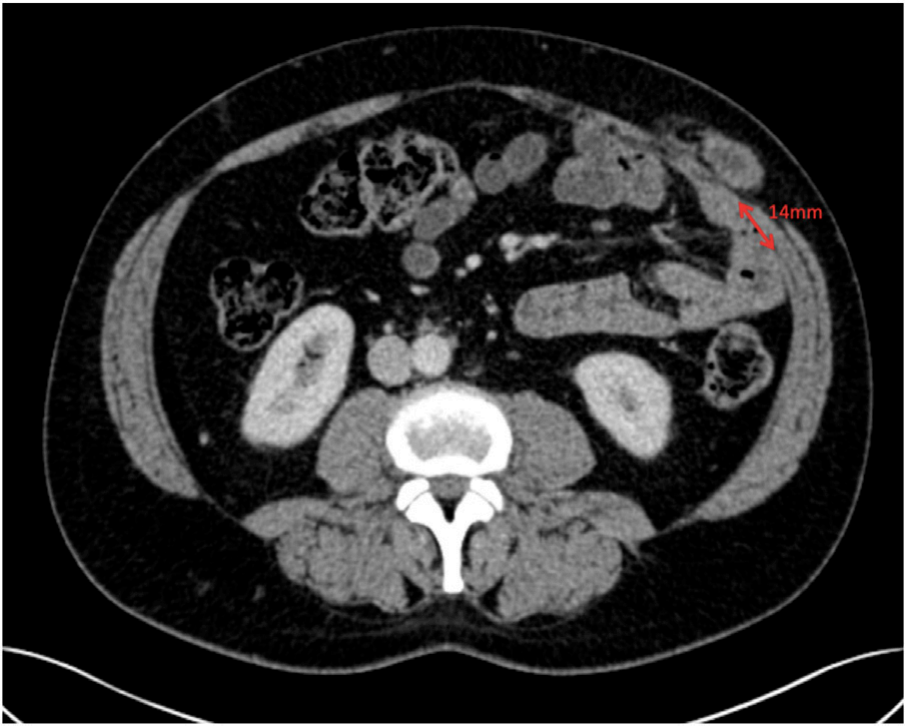
Axial image at the superior aspect of the parastomal hernia defect showing the transversus abdominis 14 mm away from the linea semilunaris.

**FIGURE 11:**
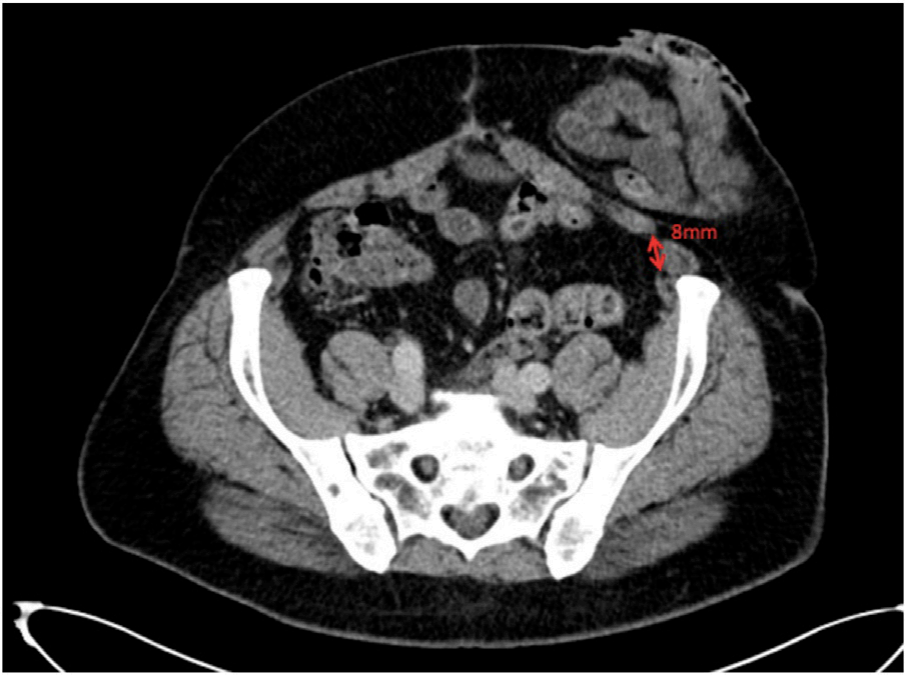
Axial image at the inferior aspect of the parastomal hernia defect showing the transversus abdominis 8 mm away from the linea semilunaris.

With anatomical variation in mind, our pre-operative CT analysis during MDT discussions have now become even more detailed, particularly when planning repairs of parastomal hernias or flank hernias using the Pauli [16] or retromuscular approaches, respectively. Knowing the location of the lateral border of rectus abdominis and the medial border of transversus abdominis in relation to the ostomy site or defect gives us greater confidence in finding the preperitoneal plane. Particularly as flank hernias can cause significant anatomical distortion. For example, Figure 9, shows a parastomal hernia protruding through the abdominal wall, with rectus muscle on either side of the defect. Our pre-operative CT analysis allows us to assess the location of the medial edge of transversus muscle at both the superior-most (Figure 10) and the inferior-most (Figure 11) aspect of the lateral defect. This gives us an awareness of when we should be able to visualise the body of the transversus abdominis muscle during dissection and reassurance when the muscle is located in its predicted location. For parastomal and flank repairs, we combine the “bottom up” [13] and “top down” [2] approaches to meet laterally behind the defect in the preperitoneal plane, and this detailed description of the transversus abdominis muscle helps us to perform these techniques with greater accuracy. Furthermore, our pre-operative analysis allows us to assess the level at which the transversus abdominis muscle crosses the semilunar line and starts to overlap the posterior sheath. For flank hernias in the right or left iliac fossa a full midline incision is often not required and finding the transversus abdominis muscle to perform the top down transversus release can be difficult. Looking at the CT, we can estimate the length of the lower midline incision required in order to visualise and get access to the transversus muscle and perform a top down release.

Our study has limitations. Firstly, the level of the umbilicus does vary [17] and we have used it as a fixed reference point for our measurements. However, as mentioned previously, many incisional hernias originate from the umbilicus, either due to a failed previous umbilical hernia repair or due to an incisional hernia after previous umbilical port insertion. Consequently, a detailed understanding of abdominal wall muscle variation at this level is required. In addition, our study does not describe rectus abdominis and lateral abdominal wall muscle variation in the longitudinal axis, but purely in the transverse axis. When hernias are being repaired the defect is closed in the transverse orientation to close the width of the defect and restore the linea alba [18]. Therefore, the amount of release achieved from a component separation will be determined exclusively by transverse anatomical variation and we would not expect longitudinal variation to be relevant. Throughout the study, we also have made substantial assumptions regarding patient selection. We have assumed that abdominal wall anatomy does not vary with patient size (height and weight), abdominal cavity dimensions (length and width), gender, age, co-morbidities, and intra-abdominal pathology. It is possible that more specific patient selection criteria may show less or more anatomical variability. Further work is required in order to explore this, and to discover its impact on reconstructive options. Lastly, it is important to mention the variability that arose between our two gastrointestinal radiologists who analysed the CT scans and obtained the measurements. Possibly, a prolonged training period prior to starting the study where the two radiologists collaborated and analysed scans together could have reduced the variability. Although this was carried out on an informal basis and formalised program of shared scan assessment followed by critiquing each other’s measurement methods may have reduced variation. Furthermore, MRI cross-sectional imaging may have improved precision and reduced inter-reader variability.

In this study we describe the anatomical variability of the anterior abdominal wall musculature. As ventral hernia morphology varies considerably, we believe that a detailed understanding and analysis of the abdominal wall muscle distribution around a hernia defect is vital in planning optimal abdominal wall reconstruction. Further studies of how abdominal wall muscle anatomy varies with difference patient characteristics are warranted.

## Data Availability

The raw data supporting the conclusion of this article will be made available by the authors, without undue reservation.

## Footnotes

1 We have used the commonly used definition for the semilunar line as defined in Gray’s Anatomy “the lateral border of the rectus muscles” (Standring, S. (2020) Gray’s Anatomy, 42nd Edition. The Anatomical Basis of Clinical Practice. Elsevier, Amsterdam). A recent publication (Vierstraete M et al. EIT Ambivium, Linea Semilunaris, and Fulcrum Abdominalis. J Abdom. Wall Surg. 2023 Dec 22:2:12217) has named this structure the “EIT Ambivium” and defines the semilunar line as “the line forming and marking the transition from muscle to aponeurosis in the transversus abdominis muscle.” These new definitions may gain traction in the literature but to make this manuscript more comprehensible we have used the most well-known definition.

## References

[1] Köckerling F Sheen AJ Berrevoet F Campanelli G Cuccurullo D Fortelny R Accreditation and Certification Requirements for Hernia Centers and Surgeons: The ACCESS Project. Hernia (2019) 23(2):185–203. 10.1007/s10029-018-1873-2 30671899 PMC6456484

[2] Novitsky YW Elliott HL Orenstein SB Rosen MJ. Transversus Abdominis Muscle Release: A Novel Approach to Posterior Component Separation During Complex Abdominal Wall Reconstruction. Am J Surg (2012) 204:709–16. 10.1016/j.amjsurg.2012.02.008 22607741

[3] Malik A MacDonald ADH De Beaux AC Tulloh BR. The Peritoneal Flap Hernioplasty for Repair of Large Ventral and Incisional Hernias. Hernia (2014) 18(1):39–45. 10.1007/s10029-013-1086-7 23568492

[4] Jensen KK Henriksen NA Jorgensen LN. Endoscopic Component Separation for Ventral Hernia Causes Fewer Wound Complications Compared to Open Components Separation: A Systematic Review and Meta-Analysis. Surg Endosc Other Interv Tech (2014) 28(11):3046–52. 10.1007/s00464-014-3599-2 24942783

[5] Carbonell AM Warren JA Prabhu AS Ballecer CD Janczyk RJ Herrera J Reducing Length of Stay Using a Robotic-Assisted Approach for Retromuscular Ventral Hernia Repair: A Comparative Analysis From the Americas Hernia Society Quality Collaborative. Ann Surg (2018) 267(2):210–7. 10.1097/SLA.0000000000002244 28350568

[6] Muirhead LJ Shaw AV Kontovounisios C Warren OJ. Establishing a Robust Multidisciplinary Team Process in Complex Abdominal Wall Reconstruction Within a Colorectal Surgical Unit. Tech Coloproctol (2019) 23(4):379–83. 10.1007/s10151-019-01965-4 30989414 PMC6536468

[7] Kumar S Rao ND Parker SG Plumb A Windsor A Mallett S Are Preoperative CT Variables Associated With the Success or Failure of Subsequent Ventral Hernia Repair: Nested Case-Control Study. Eur Radiol (2022) 32:6348–54. 10.1007/s00330-022-08701-x 35348860 PMC9381620

[8] Winters H Knaapen L Buyne OR Hummelink S Ulrich DJO van Goor H Pre-Operative CT Scan Measurements for Predicting Complications in Patients Undergoing Complex Ventral Hernia Repair Using the Component Separation Technique. Hernia (2019) 23(2):347–54. 10.1007/s10029-019-01899-8 30847719 PMC6456480

[9] Schlosser K Maloney S Prasad T Colavita PD Augentein VA Heniford BT. Three-Dimensional Hernia Analysis: The Impact of Size on Surgical Outcomes. Surg Endosc (2020) 34(4):1795–801. 10.1007/s00464-019-06931-7 31236720

[10] Reinpold W Kockerling F Bittner R Conze J Fortelny R Koch A Classification of Rectus Diastasis—A Proposal by the German Hernia Society (DHG) and the International Endohernia Society (IEHS). Front Surg (2019) 6(1):1–6. 10.3389/fsurg.2019.00001 30746364 PMC6360174

[11] Monkhouse W Khalique A. Variations in the Composition of the Human Rectus Sheath: A Study of the Anterior Abdominal Wall. J Anat (1986) 145:61–6. 2962970 PMC1166492

[12] Courreges P Poddevin F Lecoutre D. Para-Umbilical Block: A New Concept for Regional Anaesthesia in Children. Paediatr Anaesth (1997) 7(3):211–4. 10.1046/j.1460-9592.1997.d01-79.x 9189966

[13] Robin-Lersundi A Hernando LB Lopez-Monclus J Cidoncha AC Mendez CSM Jimenez Cubedo E How We Do It: Down to Up Posterior Components Separation. Langenbecks Arch Surg (2018) 403(4):539–46. 10.1007/s00423-018-1655-4 29502282

[14] Kostov S Dineva S Kornovski Y Slavchev S Ivanova Y Yordanov A. Vascular Anatomy and Variations of the Anterior Abdominal Wall - Significance in Abdominal Surgery. Prague Med Rep (2023) 124(2):108–42. 10.14712/23362936.2023.9 37212131

[15] Parker SG Halligan S Liang M Muysoms FE Adrales GL Boutall A International Classification of Abdominal Wall Planes (ICAP) to Describe Mesh Insertion for Ventral Hernia Repair. Br J Surg (2020) 107(3):209–17. 10.1002/bjs.11400 31875954

[16] Pauli EM Juza RM Winder JS. How I Do It: Novel Parastomal Herniorraphy Utilizing Transversus Abdominis Release. Hernia (2016) 20(4):547–52. 10.1007/s10029-016-1489-3 27023876

[17] Fawkner-Corbett D Nicholson JA Bullen T Cross P Bailey D Scott M. Anatomical Variation in the Position of the Umbilicus and the Implications for Laparoscopic Surgery. Int J Surg (2010) 8(7):540. 10.1016/j.ijsu.2010.07.118

[18] Criss CN Clayton CP Krpata DM Seafler CM Lai N Fiutem J Functional Abdominal Wall Reconstruction Improves Core Physiology and Quality-Of-Life. Surgery (2014) 156(1):176–82. 10.1016/j.surg.2014.04.010 24929767

